# Application of Sigma metrics in the quality control strategies of immunology and protein analytes

**DOI:** 10.1002/jcla.24041

**Published:** 2021-10-04

**Authors:** Yanfen Luo, Xingxing Yan, Qian Xiao, Yifei Long, Jieying Pu, Qiwei Li, Yimei Cai, Yushun Chen, Hongyuan Zhang, Cha Chen, Songbang Ou

**Affiliations:** ^1^ Department of Medicine Laboratory The Second Affiliated Hospital of Guangzhou University of Chinese Medicine Guangzhou China; ^2^ Department of Medicine Laboratory Guangdong Provincial Hospital of Chinese Medicine Guangzhou China; ^3^ Reproductive center, Department of Obstetrics and Gynecology Sun Yat‐sen Memorial Hospital, Sun Yat‐sen University Guangzhou China

**Keywords:** allowable total error, immunology and protein analytes, quality goal index, Sigma Method Decision Charts, Sigma metrics

## Abstract

**Background:**

Six Sigma (6σ) is an efficient laboratory management method. We aimed to analyze the performance of immunology and protein analytes in terms of Six Sigma.

**Methods:**

Assays were evaluated for these 10 immunology and protein analytes: Immunoglobulin G (IgG), Immunoglobulin A (IgA), Immunoglobulin M (IgM), Complement 3 (C3), Complement 4 (C4), Prealbumin (PA), Rheumatoid factor (RF), Anti streptolysin O (ASO), C‐reactive protein (CRP), and Cystatin C (Cys C). The Sigma values were evaluated based on bias, four different allowable total error (TEa) and coefficient of variation (CV) at QC materials levels 1 and 2 in 2020. Sigma Method Decision Charts were established. Improvement measures of analytes with poor performance were recommended according to the quality goal index (QGI), and appropriate quality control rules were given according to the Sigma values.

**Results:**

While using the TEa_NCCL_, 90% analytes had a world‐class performance with σ>6, Cys C showed marginal performance with σ<4. While using minimum, desirable, and optimal biological variation of TEa, only three (IgG, IgM, and CRP), one (CRP), and one (CRP) analytes reached 6σ level, respectively. Based on σ_NCCL_ that is calculated from TEa_NCCL_, Sigma Method Decision Charts were constructed. For Cys C, five multi‐rules (1_3s_/2_2s_/R_4s_/4_1s_/6_X_, N = 6, R = 1, Batch length: 45) were adopted for QC management. The remaining analytes required only one QC rule (1_3s_, N = 2, R = 1, Batch length: 1000). Cys C need to improve precision (QGI = 0.12).

**Conclusions:**

The laboratories should choose appropriate TEa goals and make judicious use of Sigma metrics as a quality improvement tool.

## Graphical abstract

We first evaluated the performance of 10 immunology and protein analytes based on four different sources of TEa. Further the Sigma Method Decision Charts were constructed in terms of TEa_NCCL_, providing us with a visual view of the analytes’ performance. For Cys C with σ<6, the cause for poor performance was evaluated using QGI.

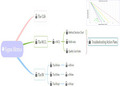

## 1 INTRODUCTION

Detection of immunology and protein analytes is widely conducted in medical laboratories in China. How to ensure test performance, provide patients with accurate and reliable results, and provide support for doctors’ diagnosis and treatment are the primary goals of medical laboratories. For this reason, Six Sigma is obviously a rare good tool for quality control (QC). Sigma, which has a statistical appellation—“Standard Deviation,” represents the data dispersion.^1^, ^2^ As we all know, the higher the Sigma value is, the better the quality is. In medical laboratories, Sigma metrics have been widely used for the quality control of the whole clinical test processes, including pre‐^3^, during‐^4^ and post‐analytical^5^ phases. A scientific and reasonable quality control strategy of medical laboratories can be achieved by combining Sigma quality management with Westgard multirules quality control charts.

Quality control is an important part of clinical laboratory management. As a commonly used quality management tool, Six Sigma management program can effectively evaluate the performance indicators of analytes, help the laboratories find problems in time. The “Six” in Six Sigma represents the ideal goal that anything beyond those tolerance specifications is considered a defect.^1^ 6σ means 3.4 defect per million with world class performance, while 3σ means 66800 defect per million with marginal performance, that is, for example, if the minimum standard of quality control is set at the 3σ level, 66800 out of 1 million human immunodeficiency virus (HIV) carriers may be misdiagnosed. Therefore, the minimum standard set at the 3σ level is not fully applicable to clinical laboratories. The laboratory needs to increase the value of Sigma, minimize those defects, and increase the probability of error detection. Six Sigma quality management provides a new perspective for the quality control strategy of clinical laboratories. Not only through Six Sigma can we identify whether our methods are appropriate for clinical but also it can help determine the QC rules, guide our risk management efforts.

In this study, we evaluated the performance of 10 immunology and protein analytes by calculating their Sigma values based on Bias%, CV%, and four different sources of TEa%. Based on the calculated Sigma value, the QC strategies were personalized and Sigma Method Decision Charts were established. Improvement measures of analytes with σ below 6 were recommended according to the quality goal index (QGI).

## 2 MATERIALS AND METHODS

### 2.1 Study design

**FIGURE 1:**
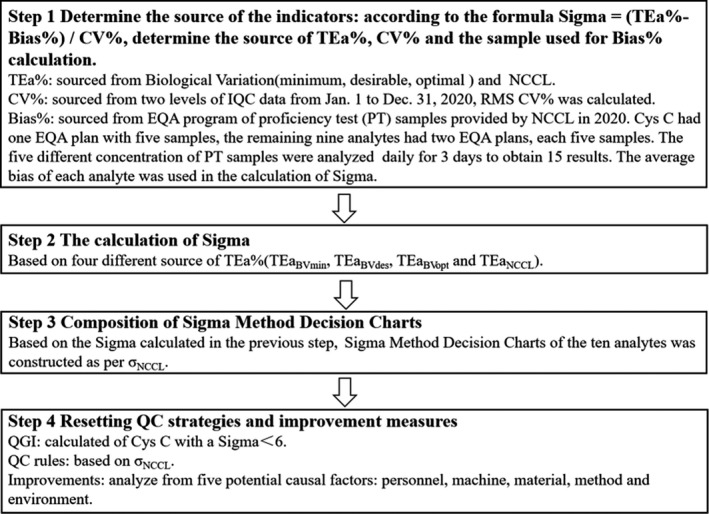
Flow chart of this research

This study included four steps (Figure 1). The study was conducted in the Laboratory Department of Guangdong Provincial Hospital of Chinese Medicine from January 1 to December 31, 2020. According to the formula Sigma = (TEa%‐Bias%)/CV%,^6^ we first determined the source of TEa%, CV%, and the sample used for Bias% calculation, second calculated the Sigma, and then Sigma Method Decision Charts were constructed, and finally the QGI analysis and corrective actions were performed to find and eliminate the potential causes of poor clinical performance of the analytes.

### 2.2 Instruments and reagents

Roche c8000 (Roche, Switzerland) automatic biochemical analysis system was used to quantify the 10 analytes: Immunoglobulin G (IgG), Immunoglobulin A (IgA), Immunoglobulin M (IgM), Complement 3 (C3), Complement 4 (C4), Prealbumin (PA), Rheumatoid factor (RF), Anti streptolysin O (ASO), C‐reactive protein (CRP), and Cystatin C (Cys C). All analytes adopted the principle of immunoturbidimetry. All the reagents and calibrators were Roche original except CRP from Sekisui (Osaka, Japan), PA from Kehua (Shanghai, China), and Cys C from Mindray (Shenzhen, China).

Two‐level internal quality controls (IQC) (Liquichek Immunology and protein Control, lot: 68931and 68932, BIO‐RAD, US) were analyzed, all controls were operated in strict accordance with the manufacturer's instructions.

Five‐level materials of external quality assessment (EQA) with different concentrations of the analytes were provided by the National Center for Clinical Laboratories (NCCL, China). Cys C (NCCL‐C‐14) had only one EQA plan in year 2020 with lots of 202011, 202012, 202013, 202014, and 202015. The remaining nine analytes, which was called “Special proteins plan (NCCL‐C‐06),” had two EQA plans: lots for the first EQA plan were 202011, 202012, 202013, 202014, and 202015; lots for the second EQA plan were 202021, 202022, 202023, 202024, and 202025. Each level material was dissolved in pure water according to the manufacturer's instructions.

### 2.3 Evaluation of imprecision

Imprecision is estimated using the coefficient of variation (CV%) which is a measure of variability and indicator of random errors. The two IQC levels of cumulative coefficient of variation (CV%) were collected from the Laboratory Department of Guangdong Provincial Hospital of Chinese Medicine from January 1 to December 31, 2020. Each analyte had two concentration levels. The root mean square coefficient of variation (RMS CV%) was calculated.^7^, ^8^, ^9^ The calculation equation is: RMS CV% = [(CV_1_^2^ + CV_2_^2^) /2]^0.5^.

### 2.4 Evaluation of bias

Bias is an indicator of systematic errors. The laboratory was involved in the EQA program by analyzing five different concentration proficiency test (PT) samples provided by NCCL. PT samples were dissolved in pure water according to the NCCL’s instructions, each PT sample was tested for 3 days to obtain three results, and the mean of the three results was calculated and assigned as “Measured mean in our laboratory.” NCCL groups the submitted data according to the instruments or reagent manufacturers that participants use, and takes the ISO13528 robust average value in the group as the target mean. Seven analytes (C3, C4, IgG, IgM, IgA, ASO, and RF) were grouped according to the instrument, while the other three analytes (CRP, PA, and Cys C) were grouped according to reagent manufacturers due to non‐Roche reagents. The target mean assigned by NCCL of each analyte was considered as target value. Excluding unqualified PT data (Data exceeding two standard deviations of the mean), the calculation equation was as follows^10^, ^11^:

Bias%=│Measured mean in our laboratory‐Target mean assigned by NCCL│/(Target mean assigned by NCCL)×100%.

The average bias of each analyte, which was calculated by 1‐year accumulative bias of each analyte sourced from the NCCL plans in 2020, was used in the calculation of Sigma.

### 2.5 Allowable total error

TEa (or “total allowable variation”) represents the allowable difference between measured value and trueness. Four different TEa targets were used in this study: (Ⅰ) TEa derived from the quality goals issued by the China National Center for Clinical Laboratories (NCCL) in 2017,^12^ designated as TEa_NCCL_. (Ⅱ, Ⅲ, Ⅳ) the biological variation database specifications (minimum, desirable, optimal), designated as TEa_BVmin_, TEa_BVdes_ and TEa_BVopt_. BV provided by EFLM.

(https://biologicalvariation.eu/) ^13^ was first adopted, secondly from other researches which EFLM does not cover (RF^14^ and PA^15^). The TEa_BV_ are calculated using the formula:

$${\text{TEa}}_{\text{BVmin}}=1.65\times 0.{\text{75CV}}_{I}+0.375{\left({\text{CV}}_{I}^{2}+{\text{CV}}_{G}^{2}\right)}^{0.5},$$

$${\text{TEa}}_{\text{BVdes}}=1.65\times 0.{\text{5CV}}_{I}+0.25{\left({\text{CV}}_{I}^{2}+{\text{CV}}_{G}^{2}\right)}^{0.5},$$

$${\text{TEa}}_{\text{BVopt}}=1.65\times 0.{\text{25CV}}_{I}+0.125{\left({\text{CV}}_{I}^{2}+{\text{CV}}_{G}^{2}\right)}^{0.5}.$$

Here CV_I_ means CV within‐subject, CV_G_ means CV between‐subject.

### 2.6 Sigma metrics calculation

Sigma value is calculated using the standard equation: Sigma = (TEa%‐Bias%)/CV%.^6^ The performance evaluation standards are divided into 6 levels: world‐class: σ≥6; excellent: 5≤σ<6; good: 4≤σ<5; marginal: 3≤σ<4; poor: 2≤σ<3; Unacceptable: σ<2. 3σ was considered as the minimum acceptable limit. The quality control rules were selected as per Westgard Sigma multi‐rules (shown in Table 1) based on Sigma value.

**TABLE 1:** Westgard Sigma multi‐rules

Sigma value	Rules adopted
σ≥6	1_3s_ (N = 2, R = 1, Batch length: 1000)
5≤σ<6	1_3s_/2_2s_/R_4s_ (N = 2, R = 1, Batch length: 450)
4≤σ<5	1_3s_/2_2s_/R_4s_/4_1s_ (N = 4, R = 1, Batch length: 200)
3≤σ<4	1_3s_/2_2s_/R_4s_/4_1s_/6_X_ (N = 6,R = 1, Batch length: 45)

Note

N, the number of quality control determinations per batch, N = 2, represented two measurements of a single QC level or one measurement of two QC levels, N = 2 similar definitions apply to N = 4 and N = 6. R, the number of batch. Batch length: The maximum number of samples in a round of quality control.

### 2.7 Composition of Sigma Method Decision Charts

Logging in the NCCLnet (https://www.nccl.org.cn/loginCn), entering TEa%, Bias%, and CV% of each analyte which is obtained through the above steps in the interface of the Six Sigma management menu, the Sigma Method Decision Charts are composed with CV%/TEa% along the x‐axis and Bias%/TEa% along the y‐axis.^10^, ^16^, ^17^, ^18^ Five lines divide the chart into 6 levels: world‐class (σ> 6), excellent (5≤σ< 6), good (4 ≤σ< 5), marginal (3≤σ< 4), poor (2≤σ< 3), and unacceptable (σ< 2). The specific Sigma value of the analyte is displayed as a point in the chart, providing us with a visual view of the analyte's performance.

### 2.8 Quality goal index ratio

The QGI ratio was calculated from the analyte with a Sigma<6. The calculation equation is as follows: QGI =Bias%/(1.5×CV%). A QGI value less than 0.8 (QGI <0.8) indicates that the precision needs to be improved, whereas a value greater than 1.2 (QGI >1.2) indicates that the accuracy needs to be improved. A QGI value between 0.8 and 1.2 (0.8 ≤ QGI ≤1.2) indicates that both accuracy and precision need to be improved. Analytes with lower Sigma were analyzed according to QGI, that is, the problem was due to precision or accuracy or combination of both, helping the laboratories to take targeted measures.^10^, ^16^, ^17^, ^18^

## 3 RESULTS

### 3.1 Cumulative Mean, SD, CV%, Bias%, and TEa% derived from four standards for 10 immunology and protein analytes

Cumulative Mean, SD, and CV% of two IQC levels were shown in Table 2. The RMS CV% ranged from 1.83% (IgG) to 4.96% (Cys C). The EQA data, which were all falling within ±2SD of the mean, were all satisfactory. The average bias values were displayed in Table 2. They ranged from 0.93% (Cys C) to 4.42% (C3). Cys C had the highest CV% and lowest Bias%. Four different source of TEa were also shown in Table 2. Mean assigned by NCCL and relative bias% were shown in Table S1.

**TABLE 2:** Mean, SD, RMS CV%, Bias% and TEa% derived from four standards for 10 analytes

Analyte	QC level	Cumulative Mean	SD	CV%	RMS CV%	Bias%	TEa%			
							TEa _BVmin_	TEa _BVdes_	TEa _BVopt_	TEa _NCCL_
IgG	level 1	7.76	0.12	1.55	1.83	1.97	10.88	7.25	3.63	25
	level 2	12.9	0.27	2.07						
IgA	level 1	1.1	0.02	1.82	1.98	2.41	14.67	9.78	4.89	25
	level 2	2.36	0.05	2.13						
IgM	level 1	0.54	0.01	2.7	2.29	4.02	25.62	17.08	8.54	25
	level 2	1.11	0.02	1.79						
C3	level 1	0.85	0.02	2.38	2.98	4.42	11.65	7.77	3.88	25
	level 2	1.8	0.06	3.47						
C4	level 1	0.13	0.00	3.03	3.13	1.60	18.08	12.06	6.03	25
	level 2	0.3	0.01	3.23						
CRP	level 1	10.21	0.35	3.43	2.81	4.22	76.06	50.70	25.35	25
	level 2	26	0.53	2.02						
RF	level 1	21	0.66	3.13	3.23	3.83	20.25	13.50	6.75	25
	level 2	31	1.03	3.33						
PA	level 1	143	5.15	3.6	3.60	1.14	21.75	14.50	7.25	25
	level 2	256	9.19	3.59						
Cys C	level 1	0.41	0.02	4.84	4.96	0.93	9.73	6.49	3.24	20
	level 2	0.57	0.03	5.08						
ASO	level 1	87	3.04	3.49	2.83	2.12	‐^†^	‐^†^	‐^†^	25
	level 2	143	2.79	1.95						

Abbreviations: Cumulative Mean, the concentration of two levels of IQC; SD, Standard Deviation; CV%, Cumulative coefficient of variation; RMS CV%, Root Mean Square coefficient of variation; TEa_BVmin_, TEa_BVdes_, TEa_BVopt_, represented the TEa sourced from the minimum, desirable, and optimal biological variation database specifications, respectively; TEa_NCCL_ represented the TEa sourced from the NCCL.

† The TEa source did not cover the TEa of the analyte.

### 3.2 Distribution of Sigma metrics based on four TEa standards

Table 3 showed the Sigma values of 10 immunology and protein analytes based on four different TEa standards. While using the TEa_NCCL_, 90% (9/10) analytes had a world‐class performance with σ>6, Cys C showed marginal performance with σ<4. However, while we chose the TEa based on biological variation database specifications, the Sigma values were not so satisfactory. Since the TEa_BVmin_ of only two analytes IgM and CRP were looser than TEa_NCCL_, σ_BVmin_ of IgM, and CRP were greater than σ_NCCL_. The other 8 analytes had lower σ_BVmin_ than σ_NCCL_. Using minimum biological variation of TEa, seven of the nine analytes (excluded ASO) exhibited a performance of at least 3σ (marginal),and three of these analytes (IgG, IgM, and CRP) had a world‐class performance. While using desirable and optimal biological variation of TEa, only one analytes (CRP) reached 6σ level, with poor or unacceptable Sigma values of the remaining nine analytes. TEa_BV_ is too strict for our laboratory, we chose the TEa_NCCL_ specifications for the follow‐up analysis, such as QGI analysis, the QC strategies construction, and composition of Sigma Method Decision Charts.

**TABLE 3:** Sigma of 10 analytes based on four different TEa standards

Analyte	σ_BVmin_	σ_BVdes_	σ_BVopt_	σ_NCCL_
IgG	4.87	2.89	0.91	12.59
IgA	6.19	3.72	1.25	11.40
IgM	9.43	5.70	1.97	9.16
C3	2.43	1.12	−0.18	6.92
C4	5.26	3.34	1.41	7.47
CRP	25.57	16.54	7.52	7.40
RF	5.08	2.99	0.90	6.55
PA	5.73	3.72	1.70	6.64
Cys C	1.77	1.12	0.47	3.84
ASO	‐^‡^	‐^‡^	‐^‡^	8.09

Note

σ_BVmin_, σ_BVdes_, σ_BVopt_, represented the calculated Sigma sourced from the minimum, desirable, and optimal biological variation database specifications, respectively; σ_NCCL_ represented the calculated Sigma sourced from the NCCL.

^‡^ No calculated Sigma obtained.

### 3.3 Composition of Sigma Method Decision Charts

**FIGURE 2:**
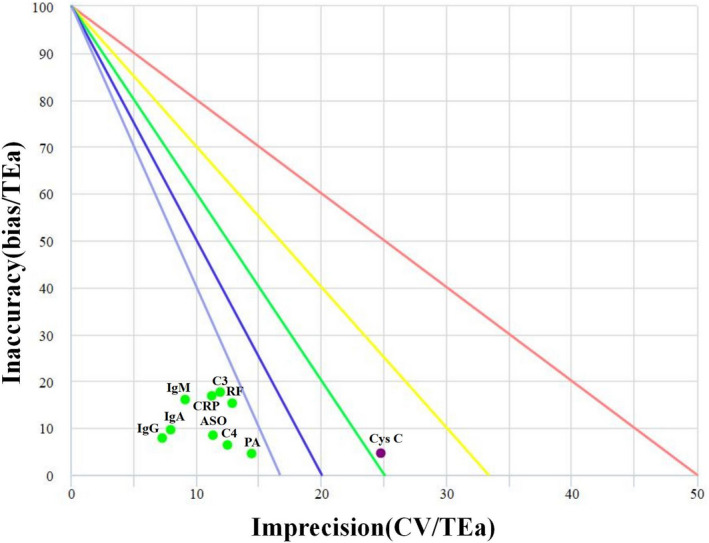
Sigma Method Decision Charts of 10 analytes based on TEa_NCCL_. The chart was drawn with CV/TEa along the x‐axis and Bias/TEa along the y‐axis, which divided into six zones by five performance lines. The zones from bottom left to top right were: world‐class (σ> 6), excellent (5≤σ< 6), good (4 ≤σ< 5), marginal (3≤σ< 4), poor (2≤σ< 3), and unacceptable (σ< 2). Different colored dots indicated different Sigma levels

We constructed Sigma Method Decision Charts of the ten analytes as per TEa_NCCL_ (Figure 2). Nine out of ten analytes were displayed in the Six Sigma zone, while Cys C appeared in the 3σ‐ 4σ zone. This chart could provide us with a visual view of the analytes' performance. We could intuitively judge the performance of the analytes through this chart.

### 3.4 Resetting QC strategies and improvement measures

QC strategies were reset according to σ_NCCL_ and the QGI of analytes with σ<6 were calculated (Table 4). For analytes with σ>6 (world‐class performance), those were IgG, IgA, IgM, C3, C4, CRP, RF, PA, and ASO, only one QC rule (1_3s_), one measurement of two QC levels (N = 2) per QC event, and up to 1000 clinical samples in a round of quality control (Batch length: 1000) were adopted for QC management. For Cys C that had “marginal” performance, five multi‐rules (1_3s_/2_2s_/R_4s_/4_1s_/6_X_) with N = 6 and up to 45 clinical samples in a round of quality control (Batch length: 45) were adopted for QC management. The calculated QGI (0.12) showed that Cys C had low precision and required a close monitoring and troubleshooting from the aspects of personnel, equipment, material, method, and environment in the daily QC.

## 4 DISCUSSIONS

In this study, we evaluated the performance of 10 immunology and protein analytes (which named after their BIO‐RAD controls—“Immunology and Protein Control”) at the Laboratory Department of Guangdong Provincial Hospital of Chinese Medicine for a one year period based on Sigma metrics. The previous studies mainly focused on the evaluation of analytical performance of clinical biochemistry analytes,^19^, ^20^, ^21^ endocrine analytes,^10^, ^22^ tumor marker analytes,^11^, ^22^ etc. using Sigma metrics. This study is the first time to focus on the application of Sigma metrics in immunology and protein analytes (IgG, IgA, IgM, C3, C4, CRP, RF, PA, ASO, and Cys C) based on four different sources of TEa. Further the Sigma Method Decision Charts were constructed in terms of TEa_NCCL_, providing us with a visual view of the analytes’ performance. For analytes with σ<6, the cause for poor performance was evaluated using QGI similar to previous studies.^10^, ^11^

According to the equation Sigma = (TEa% ‐ Bias%)/CV%, significantly different σ values are obtained using the same Bias% and CV% but different total allowable error targets similar to the previous studies,^10^, ^11^ the greater the TEa is, the greater the Sigma value is. In this study, initially five different TEa targets were included. Most TEa selected from the Clinical Laboratory Improvements Amendments (CLIA)‐88 requirements from the United States are exhibited as 3 times the standard deviation or as semi‐quantitative results^23^ (data not shown), regardless of whether the standard deviation comes from IQC or the same group variation of the EQA, the variable standard deviations bring difficulties to calculation, and there may be an illusion that analytes with large TEa have better performance and analytes with small TEa have poor performance. Therefore, we would not include TEa_CLIA_ in subsequent calculations. The Sigma metrics of 10 immunology and protein analytes were shown in Table 3 based on four different TEa standards.

As mentioned above, surprisingly, while using the TEa_NCCL_, 9 analytes for IgG, IgA, IgM, C3, C4, CRP, RF, PA, and ASO had a world‐class performance with σ>6, Cys C showed marginal performance with σ<4. On the other hand, while we chose the TEa_BV_, the Sigma values were quite different, even negative (C3). TEa sources play an important role in the calculation of Sigma value.^24^ According to the 2015 Strategic Conference on Quality Specifications convened in Milan consensus, there are three more compact levels or models, that is clinical outcomes, biological variabilities, and state‐of‐the‐art, to determine performance specifications in clinical laboratories.^25^ The actual clinical use of the test results is the gold standard for setting TEa. The optimal TEa should be established depending on the conditions and requirements of the individual laboratory. The laboratory should assess the suitability of the TEa for clinical use and determine if that would allow a larger TEa choice. TEa_BV_ is too strict for our laboratory, after a comprehensive analysis of laboratory status, analytes’ performance, and clinical recognition, therefore, in the subsequent analysis, we chose TEa_NCCL_ as performance specifications.

After calculating the Sigma value, how can we have a more intuitive impression of these results? At this time, Sigma Method Decision Charts are on the stage, which converts all the Sigma metrics into a simple intuitive dashboard.^25^ Nine Sigma in this research were in the “bull's eye” zone, suggesting so good performance as to world‐class. Cys C fallen in the 3σ‐4σ zone, suggesting there may be more defects. The Sigma Method Decision Charts allows us to get a succinct comprehensive view of an entire instrument's performance, provide us with a visual view of the analytes’ performance. We could intuitively judge the performance of the analytes through this chart. 

**TABLE 4:** The personalized QC strategies, QGI and Prioritize improvement of 10 analytes according to σ_NCCL_

Analyte	σ_NCCL_	QC procedure	QGI	Problem
IgG	12.59	1_3s_ (N = 2, R = 1, Batch length: 1000)	‐^§^	‐^§^
IgA	11.40	1_3s_ (N = 2, R = 1, Batch length: 1000)	‐^§^	‐^§^
IgM	9.16	1_3s_ (N = 2, R = 1, Batch length: 1000)	‐^§^	‐^§^
C3	6.92	1_3s_ (N = 2, R = 1, Batch length: 1000)	‐^§^	‐^§^
C4	7.47	1_3s_ (N = 2, R = 1, Batch length: 1000)	‐^§^	‐^§^
CRP	7.40	1_3s_ (N = 2, R = 1, Batch length: 1000)	‐^§^	‐^§^
RF	6.55	1_3s_ (N = 2, R = 1, Batch length: 1000)	‐^§^	‐^§^
PA	6.64	1_3s_ (N = 2, R = 1, Batch length: 1000)	‐^§^	‐^§^
Cys C	3.84	1_3s_/2_2s_/R_4s_/4_1s_/6_X_ (N = 6,R = 1, Batch length: 45)	0.12	precision
ASO	8.09	1_3s_ (N = 2, R = 1, Batch length: 1000)	‐^§^	‐^§^

^§^ Analytes with σ>6 does not need to calculate QGI, and no need for improvement.

Sigma metrics are not just a tool to ascertain the performance of analytes but also a beacon for designing a more cost‐effective QC strategy. Through the assessment of Sigma metrics, we can specify the control rules scientifically, including the number of control materials and the necessary frequency of running those controls. The closer the operating dot of an analyte is to the chart's origin, the easier the QC program is. In our laboratory, we chose 1_3s_/2_2s_ QC procedure with two QC levels once per day for the 10 immunology and protein analytes empirically to supervise the performance of analytes in the past. By using Sigma metrics, nine analytes for σ>6 can safely use the 1_3s_ procedure with one measurement of two QC levels to gain an appropriate level of analytical quality assurance, avoiding economic costs, and overwork. On the contrary, with the decline in performance, more quality control rules, more different levels of quality controls, and higher quality control frequency are required. As Cys C in this study, that had “marginal” performance, five multi‐rules (1_3s_/2_2s_/R_4s_/4_1s_/6_X_) were needed and the QC frequency could be increased to one control per 45 clinical samples. As Cys C had 38 average daily measurements in our laboratory,one time QC frequency per day is needed.

Though the Westgard Sigma multi‐rules provide a scientific and reasonable method for setting QC procedure, it could not fully reflect the precision and accuracy of the method. QGI is a tool to provide easy insights into the reasons for quality errors such as those caused by imprecision, inaccuracy, or both.^21^, ^26^ Cys C (QGI = 0.12) had low precision and some action must be taken. We make continuous improvements from five potential causal factors: personnel, machine, material, method, and environment: (Ⅰ) Strengthen personnel training, including QC knowledge and analyzer operation training; (Ⅱ) Perform regular maintenance and calibration of analyzers to ensure the performance of the analyzer, and timely retire the analyzer with poor performance; (Ⅲ) Monitor the temperature of reagent transportation and storage, avoid reagent expiration and excessive on‐board time; (Ⅳ) Revise the operation protocols, perform the machine in strict accordance with the operation protocols; (Ⅴ) Ensure that the working environment of the instrument meets the requirements, such as temperature and humidity. In fact, Cys C used in this research is a third‐party reagent, not Roche original, the reagent batch is updated so quickly, and the difference between batches is large, resulting in a large CV. Aware of this, our laboratory try our best to use the same batch reagents. We would not replace the new batch reagents until the expiration date. In fact, combined with the increase calibration frequency, our laboratory has reduced the average CV of Cys C to below 4% from January to July 2021, leading improvement of precision. Using Westgard Advisor subfunction of BIO‐RAD Unity Real Time, Cys C has higher probability of error detection (Ped), from 0.5 using 1_3s_/2_2s_ QC procedure increased to 0.995 using 1_3s_/2_2s_/R_4s_/4_1s_/6_X_ rules, but also higher probability of false rejection (Pfr), from 0.006 using 1_3s_/2_2s_ QC procedure increased to 0.095 using 1_3s_/2_2s_/R_4s_/4_1s_/6_X_ rules. In future studies, the other aspects should be prioritized to generate more conclusive results.

Nevertheless, there were three aspects of limitations in this research as follows: (Ⅰ).

Because the target means in PT/EQA plans were derived from statistical results of peer groups without measurement traceability,^21^, ^27^ those using this approach to calculate bias should be aware of possible limitations, including statistical methods used to generate the data and the number of laboratories that participate. There may be increased imprecision due to the small number of laboratories. There also might be concerns about the commutability of PT samples,^28^ because they are not the same as real patient specimens. (Ⅱ) Another weakness is that the bias evaluation in our research (analyze PT samples at five different concentrations daily for 3 days to obtain 15 results) was not conducted strictly to the method described in the CLSI EP15‐A2 document (analyze one run per day with 3 replicate samples at each of different concentrations daily for 5 days to obtain 15 results), which may have underestimated bias.^29^ (Ⅲ) The performance specification used to benchmark the methods is the most common limitation in the Sigma researches. Even the Milan consensus details only ideal considerations, there is no one source of analytical goals for all tests, laboratories must choose appropriate practical goals for each individual analytes.

This research can provide support for laboratories to select detection systems for these 10 analytes and aid individual laboratories in their choice of proper TEa goals and in working out a detailed troubleshooting action plan as a part of their quality improvement tool. Laboratory staffs can use these tools to help them select high‐quality products, further contributing to the delivery of excellent quality healthcare for patients. The result is more efficient instrument operation, more optimized laboratory workflow, and more reliable test results, ultimately helping clinicians better diagnose and treat their patients.

## CONFLICT OF INTEREST

None declared.

## AUTHOR CONTRIBUTIONS

YL and SO designed the study, drafted the work, analyzed and interpreted the data, and wrote this article; XY, QX, YL, and JP searched the literature, performed the experimental procedure; QL, YC, YC, and HZ supervised this study and reviewed this article; CC reviewed this article. All authors have read and approved the final manuscript.

## Supporting information

Table S1

Supplementary Material

## Data Availability

Some or all data generated or used during the study are available on NCCLnet (https://www.nccl.org.cn/mainCn), the Login account and password are private that cannot be shared. The relevant data in the article are available from the corresponding author (Email: ousongbang@126.com ) by request.
