# Supplementary material for: Application of Sigma metrics in the quality control strategies of immunology and protein analytes
Source: J Clin Lab Anal. 2021 Oct 4;35(11):e24041. doi: 10.1002/jcla.24041 (PMC8605144; doi:10.1002/jcla.24041)
Supplement: Supplementary file 1 — Table S1 [file JCLA-35-e24041-s001.doc]

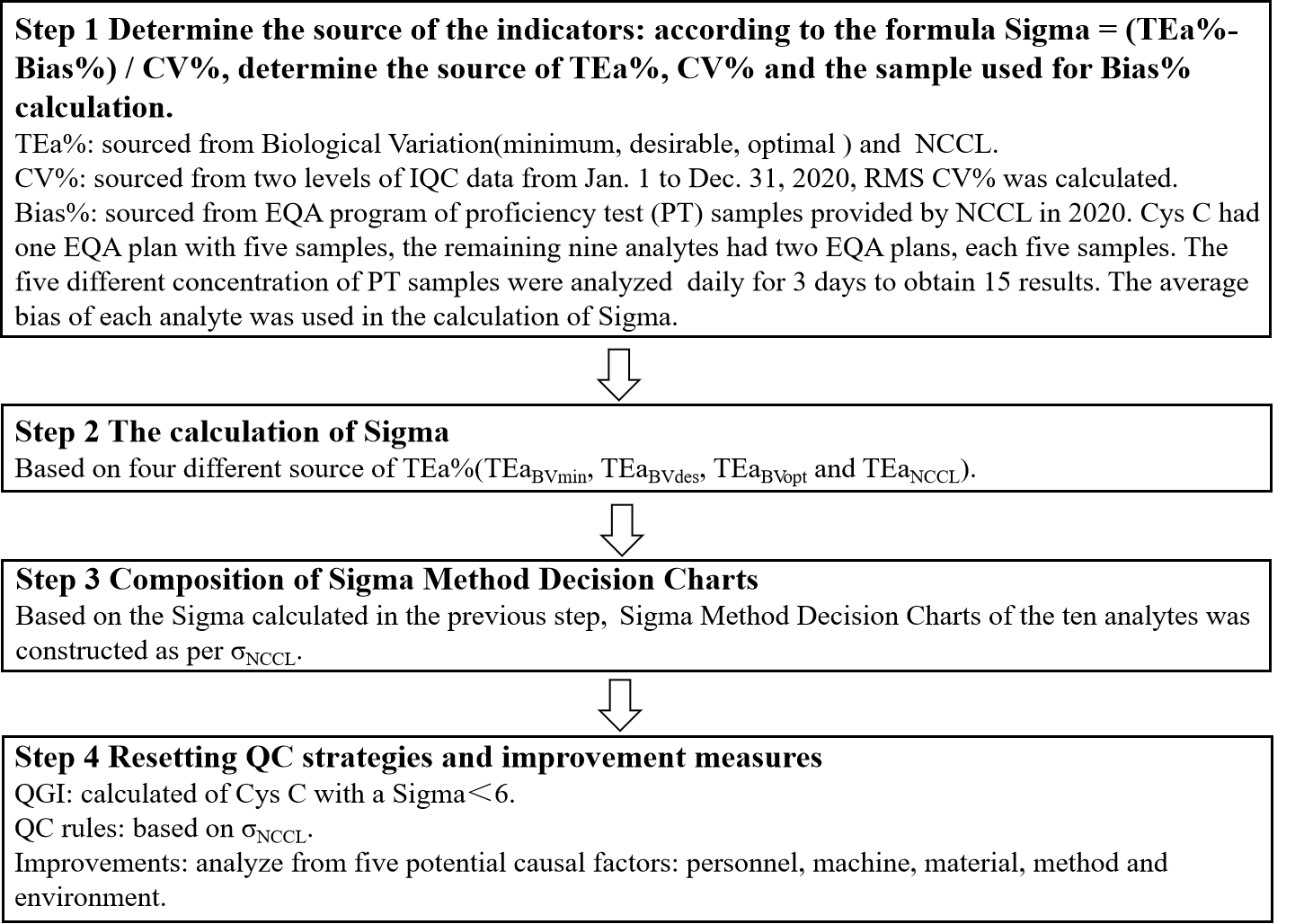


|  | |
| --- | --- |
|  |  |
|  |  |
|  |  |
|  |  |
|  |  |

10 analytes

|  |  |  |  |  |  |  |  | | | |
| --- | --- | --- | --- | --- | --- | --- | --- | --- | --- | --- |
|  |  |  |  |
|  |  |  |  |  |  |  |  |  |  |  |
|  |  |  |  |
|  |  |  |  |  |  |  |  |  |  |  |
|  |  |  |  |
|  |  |  |  |  |  |  |  |  |  |  |
|  |  |  |  |
|  |  |  |  |  |  |  |  |  |  |  |
|  |  |  |  |
|  |  |  |  |  |  |  |  |  |  |  |
|  |  |  |  |
|  |  |  |  |  |  |  |  |  |  |  |
|  |  |  |  |
|  |  |  |  |  |  |  |  |  |  |  |
|  |  |  |  |
|  |  |  |  |  |  |  |  |  |  |  |
|  |  |  |  |
|  |  |  |  |  |  |  |  |  |  |  |
|  |  |  |  |
|  |  |  |  |  |  |  |  |  |  |  |
|  |  |  |  |

|  |  |  |  |  |
| --- | --- | --- | --- | --- |
|  |  |  |  |  |
|  |  |  |  |  |
|  |  |  |  |  |
|  |  |  |  |  |
|  |  |  |  |  |
|  |  |  |  |  |
|  |  |  |  |  |
|  |  |  |  |  |
|  |  |  |  |  |
|  |  |  |  |  |

|  |  |  |  |  |
| --- | --- | --- | --- | --- |
|  |  |  |  |  |
|  |  |  |  |  |
|  |  |  |  |  |
|  |  |  |  |  |
|  |  |  |  |  |
|  |  |  |  |  |
|  |  |  |  |  |
|  |  |  |  |  |
|  |  |  |  |  |
|  |  |  |  |  |

Supplementary table

Table S1 Mean assigned by NCCL and relative bias%

| Analyte |  | PT lots | | | | | | | | | |
| --- | --- | --- | --- | --- | --- | --- | --- | --- | --- | --- | --- |
|  |  | 202011 | 202012 | 202013 | 202014 | 202015 | 202021 | 202022 | 202023 | 202024 | 202025 |
| IgG | Mean_NCCL_ | 8.24 | 14.699 | 10.926 | 18.306 | 13.062 | 11.033 | 19.472 | 12.902 | 9.541 | 16.072 |
|  | Bias% | 2.06 | 0.47 | 1.06 | 1.11 | 2.9 | 2.78 | 4.63 | 0.25 | 2.72 | 1.67 |
| IgA | Mean_NCCL_ | 1.161 | 2.123 | 1.676 | 2.548 | 1.909 | 1.779 | 2.48 | 2.03 | 1.598 | 2.245 |
|  | Bias% | 2.67 | 2.45 | 2.75 | 1.1 | 1.62 | 1.74 | 3.23 | 4.43 | 0.75 | 3.34 |
| IgM | Mean_NCCL_ | 0.555 | 1.042 | 0.807 | 1.266 | 0.932 | 0.868 | 1.243 | 0.994 | 0.761 | 1.110 |
|  | Bias% | 4.5 | 3.65 | 5.39 | 3.48 | 7.3 | 4.84 | 2.17 | 0.6 | 6.44 | 1.8 |
| C3 | Mean_NCCL_ | 0.786 | 1.425 | 1.126 | 1.716 | 1.278 | 1.197 | 1.676 | 1.369 | 1.073 | 1.512 |
|  | Bias% | 6.87 | 5.97 | 6.57 | 5.48 | 11.11 | 2.76 | 0.36 | 2.26 | 0.28 | 2.51 |
| C4 | Mean_NCCL_ | 0.13 | 0.25 | 0.192 | 0.297 | 0.221 | 0.204 | 0.289 | 0.235 | 0.183 | 0.261 |
|  | Bias% | 0 | 2.04 | 1.04 | 2.36 | 4.07 | 1.96 | 0.35 | 2.13 | 1.64 | 0.38 |
| CRP | Mean_NCCL_ | 17.062 | 67.11 | 33.861 | 88.539 | 53.943 | 38.609 | 92.165 | 56.167 | 15.552 | 68.86 |
|  | Bias% | 6.08 | 3.00 | 3.13 | 3.21 | 1.93 | 5.98 | 6.26 | 6.17 | 2.24 | 4.23 |
| RF | Mean_NCCL_ | 22.97 | 76.48 | 44.38 | 101.58 | 61.11 | 47.16 | 102.98 | 63.92 | 24.33 | 81.39 |
|  | Bias% | 4.48 | 3.29 | 3.65 | 2.38 | 4.73 | 3.9 | 3.9 | 4.82 | 2.75 | 4.44 |
| PA | Mean_NCCL_ | 153.7 | 286.50 | 226.6 | 344.1 | 258 | 239.6 | 340.8 | 273.7 | 211.7 | 305.2 |
|  | Bias% | 1.76 | 0.87 | 3.8 | 0.84 | 0.39 | 0.67 | 0.82 | 0.26 | 1.28 | 0.72 |
| Cys C | Mean_NCCL_ | 1.663 | 3.961 | 3.225 | 5.025 | 0.922 | -^¶^ | -^¶^ | -^¶^ | -^¶^ | -^¶^ |
|  | Bias% | 0.18 | 2.30 | 0.47 | 1.49 | 0.22 | -^¶^ | -^¶^ | -^¶^ | -^¶^ | -^¶^ |
| ASO | Mean_NCCL_ | 134.11 | 251.65 | 184.35 | 310.77 | 221.89 | 191.04 | 334.25 | 225.16 | 164.83 | 279.64 |
|  | Bias% | 2.9 | 0.93 | 1.27 | 0.4 | 2.75 | 0.5 | 0.07 | 6.15 | 4.35 | 1.92 |

^¶^: Cys C did not have the second EQA plan in 2020.
