# Supplementary figures and images for: Application of Sigma metrics in the quality control strategies of immunology and protein analytes
Source: J Clin Lab Anal. 2021 Oct 4;35(11):e24041. doi: 10.1002/jcla.24041 (PMC8605144; doi:10.1002/jcla.24041)

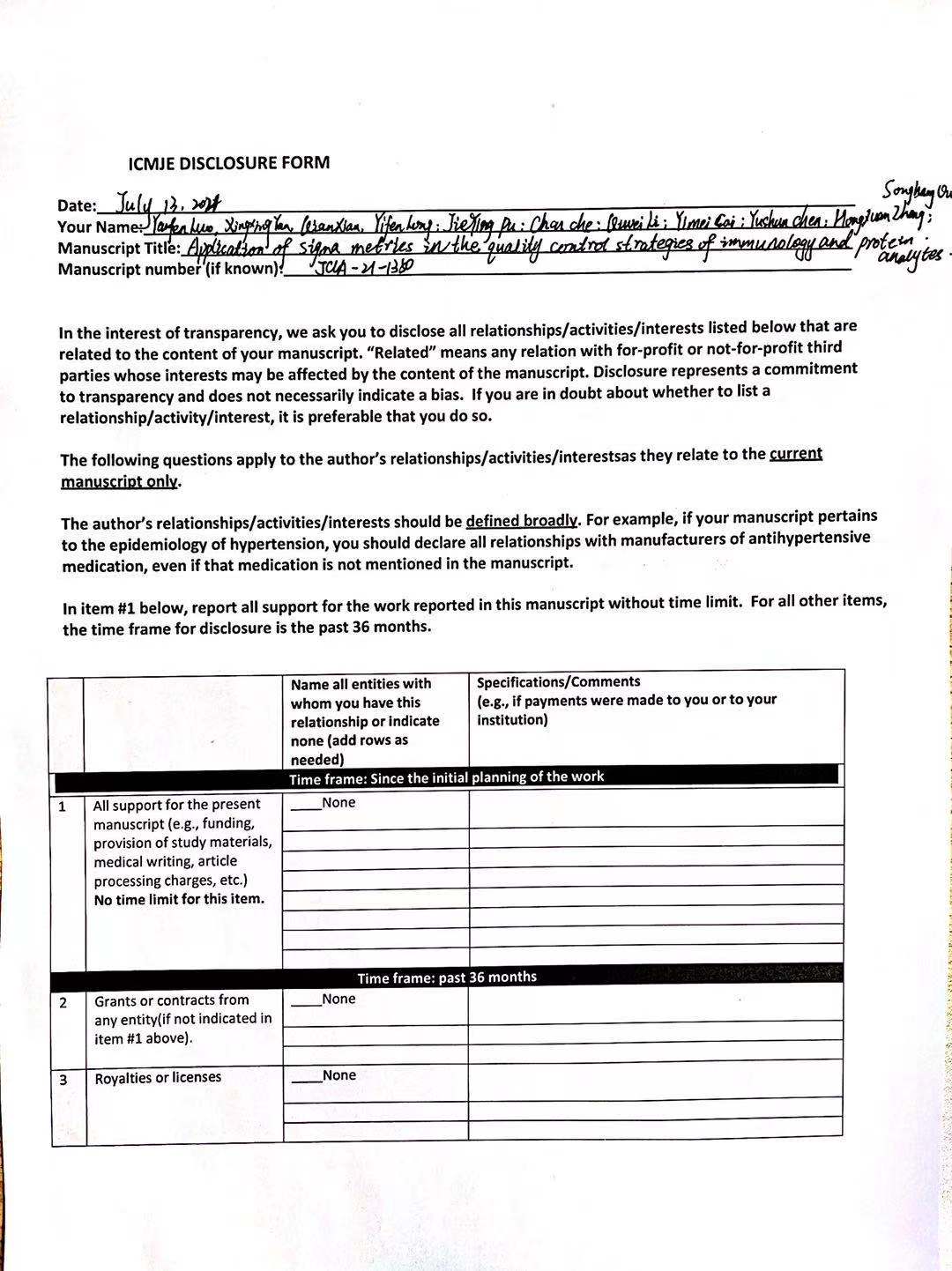


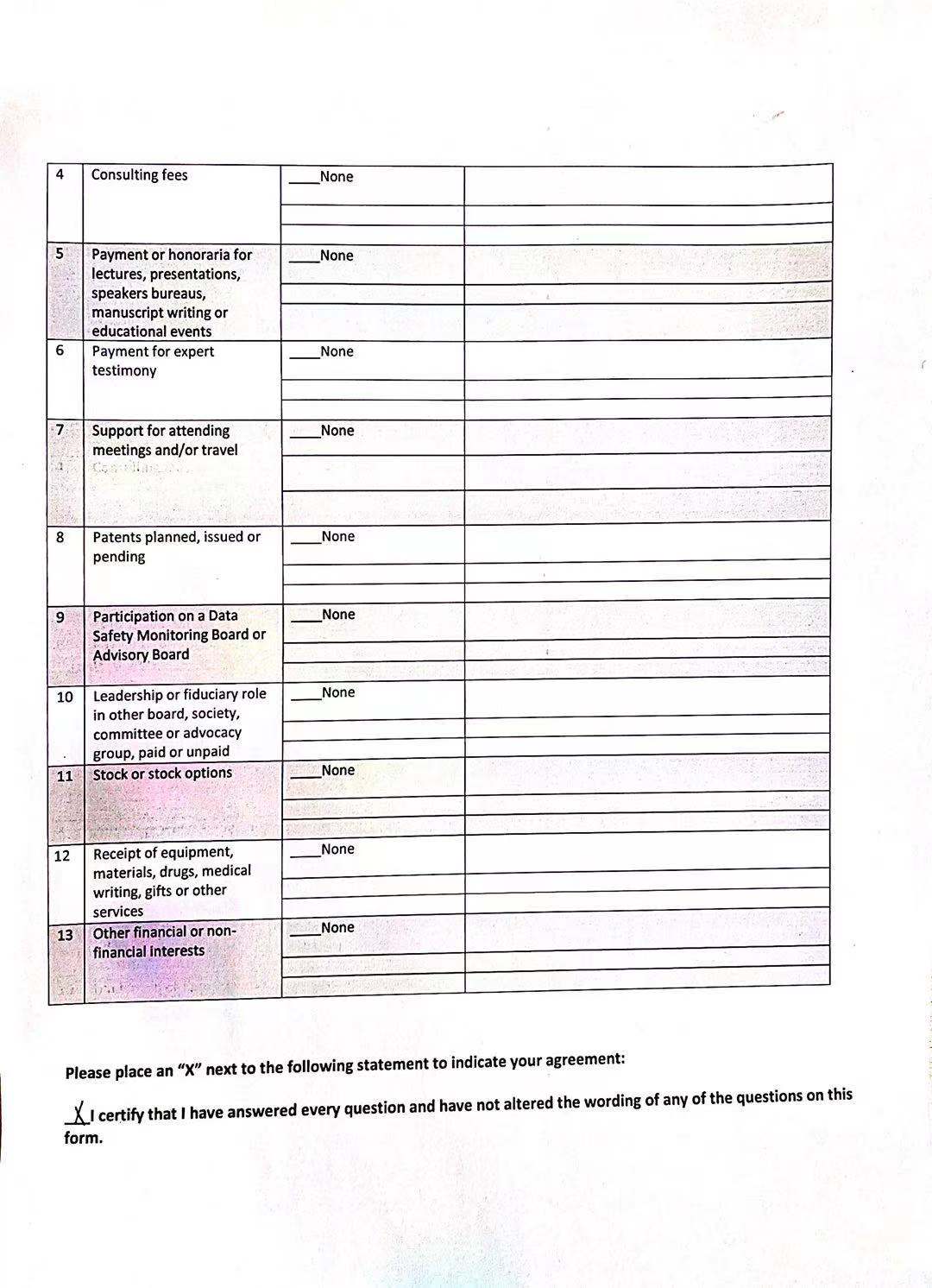

Supplement: Supplementary file 2 — Supplementary Material [file JCLA-35-e24041-s002.doc]
